# Influence of Calcium Silicate on the Chemical Properties of *Pleurotus ostreatus* var. florida (Jacq.) P. Kumm

**DOI:** 10.3390/jof6040299

**Published:** 2020-11-19

**Authors:** Rossana V. C. Cardoso, Márcio Carocho, Ângela Fernandes, Diego Cunha Zied, Juan Diego Valenzuela Cobos, Ana M. González-Paramás, Isabel C. F. R. Ferreira, Lillian Barros

**Affiliations:** 1 Centro de Investigação de Montanha (CIMO), Instituto Politécnico de Bragança, Campus de Santa Apolónia, 5300-253 Bragança, Portugal; rossana@ipb.pt (R.V.C.C.); mcarocho@ipb.pt (M.C.); afeitor@ipb.pt (Â.F.); 2 Grupo de Investigación en Polifenoles (GIP), Unidad de Nutrición y Bromatología, Facultad de Farmacia, Universidad de Salamanca, Campus Miguel de Unamuno, 37007 Salamanca, Spain; paramas@usal.es; 3 Faculdade de Ciências Agrárias e Tecnológicas (FCAT), Universidade Estadual Paulista, Campus Dracena, 17900-000 Dracena, Brazil; dczied@gmail.com; 4 Unidad Profesional Interdisciplinaria de Biotecnología (UPIBI), Instituto Politécnico Nacional, 07340 Ciudad de México CP, Mexico; juan_diegova@hotmail.com

**Keywords:** *Pleurotus ostreatus* var. florida (Jacq.) P. Kumm, substrate supplementation, harvest, biological production, chemical composition

## Abstract

Supplementation of mushroom substrates has been linked to a higher resistance against insect pests, although few studies show the impact of this supplementation on the different agronomical parameters of mushrooms or even their chemical composition. In this work, the variation in the biological and chemical composition of oyster mushroom (*Pleurotus ostreatus* var. florida (Jacq.) P. Kumm) was analysed after varying the substrate supplementation of calcium silicate (0, 0.5, 1, 2, and 4% (*w*/*w*%)) during two harvest flushes. Overall, supplementation did not change the weight, the number of fruiting bodies, biological efficiency, yield ratio, and productivity rate of the mushrooms, although the harvest flushes did show significant differences. Furthermore, slight changes were found in the chemical composition with an increase in vitamin D_2_ and tocopherols for the mushrooms with higher amounts of calcium silicate. Overall, the substrate supplementation did not seem to induce expressive changes or decrease production yields, and can, therefore, continue to be researched as a potential application to fight agronomical pests.

## 1. Introduction

Mushrooms have been a highly appreciated food since ancient times due to their nutritional value and medicinal properties. They are rich sources of compounds, namely, ergosterol (precursor of vitamin D_2_), phenolic compounds, tocopherols, ascorbic acid, and carotenoids, responsible for the bioactive properties attributed to mushrooms [1].

An edible mushroom that has attracted quite a lot of interest in recent years is *Pleurotus ostreatus* (Jacq.) P. Kumm. (pearl oyster mushroom, tree oyster mushroom) due to the ease of its cultivation, great economic potential, and flavor [2,3]. There is, therefore, the need to further study its composition in bioactive compounds to better understand their health benefits, but also increase crop yields, thus increasing its production.

One variety of the oyster mushroom, the florida variety, also known as “Hiratake” is among the most consumed in the world, with its benefits being widely described [4,5]. Royse et al. [6] considered these species the second most popular cultivated mushroom of the last decades behind shiitake mushroom (*Lentinula edodes*), due to the easiness of its cultivation which can be achieved using lignocellulosic wastes.

Silicon (Si) is the second most abundant element in the earth’s crust and its effect on organisms may be different, serving as a cellular chemical element or providing a natural morphological modification [7,8,9,10,11]. As a cellular chemical element, it plays an important role in the mineral nutrition of plants, aiding development by increasing the organism’s biomass. Furthermore, it also increases resistance to biotic and abiotic stresses, such as diseases and pests, excess of toxic metals, saline stress, water deficiency, among others [7]. Some studies indicate that Si affects the nutritional status of crops, and thus, it is believed that Si influences nutrient absorption and nutritional efficiency of plants.

On the other hand, calcium silicate is also used to provide a natural morphological modification in insects, that is, when ingested occurs damage to the oral apparatus of pests that attack plants, due to its crystalline physical structure [9,12,13,14,15,16]. Previous studies have reported reduced insect feeding on turf treated with calcium silicate, as well as others that show that calcium silicate enhances resistance of sugarcane to the African talk borer *Eldana saccharina* [13,17].

Over the past 20 years, the benefits of Si supplementation in crops have been disseminated, however, much of this information has not been consolidated, and to the authors’ best knowledge, there are very few studies on the supplementation of Si in oyster mushroom cultivation. One report on the use of Si on oyster mushrooms was published by Thongsook and Kongbangkerd [8] and described the influence of calcium and silicon on the biological and production yields of *P. ostreatus*.

In this work, the aim was to verify the effect of the crystalline structure on the cell expansion of the fungus, providing an increase for ergosterol inside the cells of the harvested mushrooms. Another concern was to assess the harmful effect on mushroom yield. Therefore, two strains of *Pleurotus ostreatus* var. florida were cultivated in varying concentrations of calcium silicate supplementation (0, 0.5%, 1%, 2%, and 4% (*w*/*w*)) and later harvested in two flushes, ten days apart. The mushrooms were analysed in terms of the total weight of harvested mushrooms, the number of mushrooms per flush, biological efficiency, yield ratio, and productivity rate. Furthermore, the chemical composition was analysed for the following groups of compounds: soluble sugars, tocopherols, individual fatty acids, ergosterol, and its derivative, vitamin D_2_. The main goal was to verify the influence of substrate supplementation with Si on the viability of the mushrooms, but also the chemical profile.

## 2. Materials and Methods

### 2.1. Chemicals and Reagents

Acetonitrile 99.9%, n-hexane 95%, and ethyl acetate 99.8% of HPLC grade were purchased from Fisher Scientific (Lisbon, Portugal). The fatty acids methyl ester (FAME) reference standard mixture 37 (standard 47885-U) was purchased from Sigma (St. Louis, MO, USA), as also other individual fatty acid isomers, Trolox (6-hydroxy-2,5,7,8-tetramethylchroman-2-carboxylic acid), sugar (D(-)-fructose, D(-)-mannitol, and D(+)-trehalose), tocopherol (α-, β-, and γ-isoforms), while Racemic tocol, 50 mg/mL, was purchased from Matreya (Pleasant Gap, PA, USA). Dimethylsulfoxide (DMSO) (Fisher Scientific, Loughborough, UK) was used as a solvent in ergosterol and vitamin D_2_ assays. Methanol, ethanol, hexane, and all other chemicals were of analytical grade and purchased from common sources. Water was treated in a Milli-Q water purification system (TGI Pure Water Systems, Greenville, SC, USA).

### 2.2. Mushroom Strains

The strains of *Pleurotus ostreatus* var. florida (Jacq.) P. Kumm used in this study were collected from producers in the city of Mogi-das-Cruzes (coordinates: –23.52327, –46.2659766) henceforth described as MC (Funghi & Flora company, Valinhos City, Sao Paulo State, Brazil, codified as FF 20), and another in the city of Presidente Prudente (–21.9629448, –51.6337427), described as PP (Brasmicel company, Suzano City, Sao Paulo State, Brazil, codified as PF 14), both in the state of São Paulo in Brazil. The samples had been identified and their strains were deposited with the following codifications (POS 16/01 and POS 16/02) at the “Centro de Estudos em Cogumelos (CECOG)”, Faculty of Agrarian and Technologic Sciences of the São Paulo State University (Universidade Estadual Paulista–UNESP, Sao Paulo State, Brazil), at Campus Dracena in the city of Dracena [18]. The inoculum was prepared by subculturing the mushroom, following the steps of production of subculture (petri dish with potato dextrose agar (PDA)), production of mother spawn, and production of grain spawn (sorghum with lime and gypsum) for compost inoculation, as described by Andrade et al. [19].

### 2.3. Substrate and Supplementation

The substrate was made using a mixture of sugarcane bagasse (500 kg), *Brachiaria dictyoneura* (1000 kg), rice bran (100 kg) and wheat bran (100 kg), calcitic limestone (50 kg), and gypsum (50 kg), as described by Zied et al. [20]. During phase I of the process, *B. dictyoneura* and sugarcane bagasse (bulk material) were moistened for 2 days. On the 3rd day, the pile was assembled, and on the 4th day, the pile was turned and, then, the additional materials (rice and wheat bran, calcitic limestone, and gypsum) were added. Afterward, two more turns were performed and on the 7th day, the substrate was transferred to a pasteurization chamber (phase II). The substrate was pasteurized between 65 and 72 °C for 20 h and subsequently conditioned between 55 and 48 °C for 1 day.

After pasteurization and conditioning, with the substrate at ambient temperature, the strains (PP and MC) were inoculated (2% of the substrate’s wet weight) together with the addition of calcium silicate (concentration of 0.5, 1, 2, and 4% substrate’s wet weight), in plastic bags containing 2 kg of wet substrate, following supplementation methodologies during spawning [21,22,23]. A control treatment was used as a reference (without the application of calcium silicate) to verify the viability of the technique. Each treatment had 5 repetitions, represented by bags of 2 kg of the substrate. The chemical-physical composition of the substrate used was N content—1.03%, C/N ratio—63/1, pH—6.7, and moisture—67%.

### 2.4. Growing Cycle

During the spawn run the air temperature and relative humidity were maintained at 26 ± 1.5 °C and 80%, respectively. For the induction of primordia and harvest, the temperature was reduced to 22 ± 1.5 °C while the relative humidity was increased up to 90%. The mushrooms were harvested twice (1st flush started 27 days after inoculation and 2nd flush started 39 days after inoculation) during the crop cycle, totalling 45 days of the growing cycle. The interval between flushes varied from 3 to 7 days depending on the strain and the calcium silicate percentages. After harvesting, the number of mushrooms per harvest and per supplementation was recorded, as was their weight. Prior to the chemical analysis, all the fruiting bodies (samples) were lyophilized (FreeZone 4.5 model 7750031, Labconco, Kansas, MO, USA) and reduced to a fine powder (20 mesh).

### 2.5. Influence of Calcium Silicate in Biological Production Yields

#### 2.5.1. Biological Efficiency

The biological efficiency (BE) is an important parameter to understand the capacity of the substrate to produce mushrooms, and thus, was calculated with the equation published by Thongsook and Kongbangkerd [8], for each flush:

(1) $$BE\left(\%\right)=\frac{fresh\;weight\;of\;mushrooms\;\left(g\right)}{weight\;of\;dry\;substrate\;\left(g\right)}\times 100$$

Equation (1). Biological efficiency of the mushrooms.

#### 2.5.2. Yield Ratio

The yield ratio is an analysed variable widely adopted in commercial crops, and was calculated following the equation:

(2) $$Y\left(\%\right)=\frac{fresh\;weight\;of\;mushrooms\;\left(g\right)}{fresh\;weight\;of\;substrate\;\left(g\right)}\times 100$$

Equation (2). Yield ratio of the mushrooms.

#### 2.5.3. Productivity Rate

The productivity rate provides an indication of how well the crop reacts to an input by providing outputs, thus, being a reason between BE and the precocity, namely days between inoculation and harvest:

(3) $$PR\left(\%\;per\;day\right)=\frac{biological\;efficience\;(\%)}{precocity\;(days)}$$

Equation (3). Daily productivity rate of the mushrooms.

### 2.6. Chemical Composition

#### 2.6.1. Soluble Sugars

For the analysis of soluble sugars, an extraction was performed with ethanol and water, followed by a filtration, previously described by Barros et al. [24]. Furthermore, the identification was performed on a Knauer High Performance Liquid Chromatograph (HPLC) (Knauer, Smartline system 1000) coupled with a refractive index detector (RI, Knauer, Berlin, Germany). The compounds were identified by comparing their retention times to the ones of commercial standards. The quantification was based on the RI signal response of each standard using the internal standard (IS) methodology, using raffinose as the IS. The HPLC conditions were as following: the mobile phase was a mixture of acetonitrile/water (70:30, *v/v*) with a flow rate of 1 mL/min. The chromatographic separation was achieved using a Eurospher 100-5 NH_2_ column (5 μm, 250 × 4.6 mm, Knauer, Berlin, Germany) at 35 °C. Data were analysed using Clarity 2.4 software (DataApex, Podohradska, Czech Republic) and the results were reported in g per 100 g of dry weight (dw).

#### 2.6.2. Tocopherols

All four isoforms of tocopherols were screened and, if detected, quantified through HPLC after extraction with hexane, methanol, and water, as previously described by Barros et al. [24] using tocol as an IS. Chromatographic separation was performed with a Polyamide II normal-phase column (250 × 4.6 mm; YMC Waters) operating at 35 °C, and the mobile phase employed was a mixture of n-hexane and ethyl acetate (70:30, *v/v*) at a flow rate of 1 mL/min. This analysis was done using the equipment described for the soluble sugars, although coupled to a fluorescence detector (FP-2020; Jasco, Easton, MD, USA), designed for excitation at 290 nm and emission at 330 nm. Quantification was based on IS methodology, and the compounds were identified by chromatographic comparisons with commercial standards. Data were evaluated using Clarity 2.4 software and the results were expressed in mg per 100 g dry weight (dw).

#### 2.6.3. Ergosterol and Vitamin D_2_

Ergosterol and vitamin D_2_ were determined after an extraction procedure previously described by Tsai at al. [25] with some modifications. Each sample (1 g) was extracted with 10 mL of dimethyl sulfoxide (DMSO) using an ultrasound bath (30 min at 45 °C, series LBX V05, Barcelona, Spain), followed by the addition of 10 mL of methanol/water (1:1, *v/v*) and 20 mL of hexane with re-extraction in an ultrasound bath. The samples were then centrifuged thrice (3000 rpm, 10 min, Centurion K24OR refrigerated centrifuge, West Sussex, UK), adding 20 mL of hexane, and removing the supernatant between each step. At the end of the extraction, the supernatants were pooled and dried using a rotary evaporator (40 °C, Büchi, Flawil Switzerland) and finally re-dissolved in 1 mL of methanol.

Using the HPLC described previously, and a UV detector (Knauer Smartline 2500), the identification and quantification of ergosterol and vitamin D_2_ was performed according to the procedure validated by Barreira et al. [26] using 280 nm as the preferred wavelength. Chromatographic separation was performed with an Inertsil 100A ODS-3 reverse phase column (5 μm, 250 × 4.6 mm, BGB Analytik AG, Boeckten, Switzerland) at 35 °C. The mobile phase used was a mixture of methanol:acetonitrile (70:30, *v/v*), with a flow rate of 1 mL/min. Data were analysed using Clarity 2.4 software. Ergosterol (Sigma-Aldrich, St. Louis, MO, USA) was quantified based on a calibration curve obtained with a commercial standard. Vitamin D_2_ was expressed in µg/100 g and ergosterol in mg per 100 g of mushroom, both in dw.

#### 2.6.4. Fatty Acids

Fatty acids were determined by gas chromatography (GC) coupled with a flame ionization detector (FID), after a transesterification procedure [24]. The analysis was carried out on a DANI model GC 1000 equipped with a split/splitless injector, the FID at 260 °C, and a Zebron-Kame column (30 m × 0.25 mm ID × 0.20 μm *df*, Phenomenex, Lisbon, Portugal). The initial column temperature was 100 °C, maintained for 2 min, then 10 °C/min until 140 °C, 3 °C/min until 190 °C, 30 °C/min until 260 °C and maintained for 2 min. Carrier gas flow (hydrogen) was 1.1 mL/min, measured at 100 °C. The split injection (1:50) was done at 250 °C. By comparing the relative retention times of FAME (*Fatty Acid* Methyl Esters) peaks of the samples with the standards, the fatty acid was identified. The results were recorded and processed using Clarity software (DataApex, Petrzilkova, Czechia) and fatty acids were expressed as a relative percentage.

### 2.7. Statistical Analysis

Throughout the manuscript, all data are expressed as mean ± standard deviation. Samples were analysed through two-way analysis of variance (ANOVA) with type III sums of squares, after verifying homoscedasticity through a Levene’s test. The post-hoc test used was either a Tukey’s multiple test (homoscedastic sample) or Tamhane T2 test (non-homoscedastic samples) for the different Calcium Silicate supplementation and a Student’s T test for the two harvest periods. Using a two-way ANOVA, it is possible to verify the influence of the two factors; Harvest number (HN) and calcium silicate supplementation (CS) independently from each other. If a significant interaction is detected among the two factors (HN × CS *p* < 0.05), they were evaluated simultaneously, and some tendencies can be extracted from the Estimated Marginal Means Plot (EMM). Inversely, if there is no significant interaction recorded among the two factors (HN × CS *p* > 0.05), they were analysed and classified independently using the post-hoc tests described above. Thus, the standard deviations were calculated from results obtained under different operational conditions, and should, therefore not be regarded as a measure of precision, rather as the range of the recorded values. All statistical analysis was performed using a significance of 0.05 and the SPSS software, version 25.

## 3. Results and Discussion

The focus of this work was to determine the influence of calcium silicate addition to the substrate of *Pleurotus ostreatus* var. florida mushrooms and assess its influence on biological efficiency, crop yield ratio, and chemical composition.

### 3.1. Biological Efficiency and Crop Yield Ratio

Table 1 shows five parameters of the mushroom crop with the supplementation of CS during the two harvest periods (1st and 2nd flushes), namely mushroom weight, number of mushrooms per harvest, biological efficiency, yield ratio, and productivity rate. Table 1 is divided into two sections, each one pertaining to the locations of the strains, namely “Presidente Prudente” (PP) and “Mogi-das-Cruzes” (MC). Each section is further divided into an upper and lower section, referring to the two factors, namely Harvest Number (HN) and Calcium Silicate supplementation (CS). This sectioning of the tables derives from the two-way ANOVA employed to understand the influence of each factor individually, and thus, in the upper section of Table 1, within the ranges of the means are all the values from the different CS, namely 0, 0.5, 1, 2, and 4%, and in the lower sections are all the values from harvests. This type of representation of the results allows for a much more trustworthy interpretation of the results, for each factor that caused change can be analysed independently of the influence of the other. The existence of an interaction between both factors is demonstrated by HN × CS *p* < 0.05 and thus only general conclusions can be extracted, whilst HN × CS *p* > 0.05 shows that each factor can be classified independently.

Analysing the strains from Presidente Prudente, it is clear that there was no interaction among the two factors for any of the analysed parameters. Still, no significant difference was recorded among the different supplementation concentrations (*p* > 0.05), while there is a significant difference amongst the two harvest periods (*p* < 0.05), being clear that all parameters are reduced in the second harvest when compared to the quantities of the first. The same tendencies can be observed for the strain from Mogi-das-Cruzes, with no statistical differences among the supplementation quantity of CS, but with a significant reduction from the first harvest to the second (classified with an *). One interesting conclusion that can be immediately extracted from the agronomical parameters investigated in this study is that calcium silicate supplementation of the substrate does not influence the total weight of the mushrooms or the number of mushrooms per harvest. Furthermore, the biological efficiency is also untouched as is the yield ratio and the productivity rate in either harvests (1st and 2nd flushes). Previous studies report similar findings, namely that the yield of production was not affected by the incorporation of calcium sources [8]. This is a positive effect, provided that supplementation would not be successful if it reduced any type of yield or productivity. For instance, the first harvest of Presidente Prudente saw a reduction from 167 to 110 g and a reduction of mushroom from 50 in the first harvest and 35 in the second.

The biological efficiency for Mogi-das-Cruzes was reduced from 108% in the first harvest to 84% in the second. Supplementation did not, however, excerpt a positive effect on yields, by increasing them either on the first or second harvest. Still, and as expected, the yields of the first harvest are almost always higher than the ones of the second, and the treatment of calcium silicate did not change this. The decrease in yield in the second harvest of *Pleurotus ostreatus* var. florida was reported by Thongsook et al. [8] and it’s natural in mushroom production once the substrate begins to be spent.

### 3.2. Effect of Supplementation on Individual Compounds

Table 2 shows the compounds that were detected in the chemical characterization of the mushrooms, namely ergosterol and its derivative, vitamin D_2_, along with various soluble sugars like fructose, mannitol, and trehalose, and also three tocopherols *α-, β*-, and *γ-.* Regarding ergosterol, the main sterol in mushrooms, it was found in much higher amounts than its derivative, vitamin D_2_. The amounts of these two compounds were similar in both mushroom strains. Trehalose was the highest sugar in both strains, with a maximum of 19 g/100 g while mannitol and fructose did not score over 2.8 and 0.2 g/100 g, respectively.

**Figure 1:**
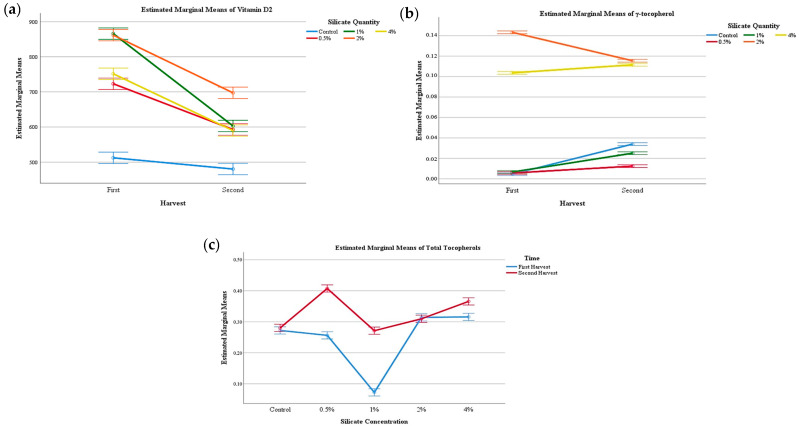
EMM plots of the Presidente Prudente mushrooms during the two harvest periods: (**a**) vitamin D_2_, (**b**) *γ*-tocopherol. EMM plots of Mogi-das-Cruzes at different Si concentrations: (**c**) total tocopherols.

Of the four tocopherol isoforms, only *δ*-tocopherol was not identified. This is not very uncommon, provided that in *Pleurotus ostreatus* var. florida mushrooms, not all isoforms are usually present [27,28]. Still, in both mushrooms, *β*-tocopherol was the most abundant. Regarding PP mushrooms, there was a significant interaction among the two factors, HN and CS, allowing for some general tendencies from the EMM plots (Figure 1). Still, regarding strains from MC, *α*-tocopherol showed that the mushrooms from the first harvest presented a statistically higher amount of this isoform than the second harvest, while all other compounds displayed a significant interaction. Some tendencies were drawn from the EMM plots that allowed for some further analysis. These results show that, once again, supplementation of the substrate with CS does not seem to alter the chemical composition of these mushrooms.

In Figure 1a the EMM plots of vitamin D_2_ of Presidente Prudente mushrooms are shown, and it is clear that there was a reduction from the first to the second harvest in terms of this vitamin. Still, the control sample showed a lower reduction but was also the sample with a lower amount of this compound to start with. Thus, supplementation of calcium silicate showed higher amounts of this vitamin, although the highest results were found at 2%. Furthermore, even at the second harvest, there was a higher amount of vitamin D_2_ than in the control samples. The fact that supplementation of calcium silicate increases the quantity of vitamin D_2_ in mushrooms is quite interesting, provided this vitamin is a beneficial compound for human health. Vitamin D comprises other vitamers beyond D_2_, although D_2_ and D_3_ are the most important ones. While D_3_ is responsible for a higher saturation of vitamin D in the human blood, it can only be produced in our skin with the help of UV light, and thus, in countries with low sun incidence, most of this vitamin enters the human body through the diet. Still, animals mainly produce D_3_, and with the tendency of the human diet to become more vegetarian based, D_2_ becomes a promising alternative, being found mainly in mushrooms and plants.

All vitamers of vitamin D are important for the human body, especially by reducing the risk of osteoporosis and other ailments of the skeleton, beyond the adjuvant effects in cancer, cardiovascular diseases, among others [29]. Interestingly, the highest amount was recorded at a 2% concentration of calcium silicate, with the 4% resulting in a reduction in vitamin D_2_. The results found for the Mogi-das-Cruzes strain show similar tendencies, with the highest amount of vitamin D_2_ found at supplementation of 2%. Thus, supplementation of the mushroom substrate with calcium silicate (2%), increases the conversion of ergosterol to vitamin D_2_ (ergocalciferol), which can be an important manner of increasing the natural production of this vitamer, becoming more available in the diet. Figure 1b shows the behaviour of *γ*-tocopherol in PP during the two harvest periods, and once again, an interesting pattern can be found, higher supplementation of calcium silicate seems to induce the production of this compound, while lower amount (up to 1%) and the control sample, although increasing from the first harvest to the second does not have as much as the 2 and 4% supplementation. The highest quantity found was for 2% supplementation at the first harvest, namely 0.13 mg/100 g, while the highest quantity in the control sample did not go over 0.035 mg/100 g, even at the second harvest. Figure 1c represents the total tocopherols of the Mogi-das-Cruzes strain for the several supplementation percentages, and once again lower supplementation of calcium silicate (1%) seems to reduce the quantity of total tocopherols, while higher amount seems to promote their production, especially 4%. Still, 0.5% CS promotes the production of tocopherols during the second harvest, which overall showed a higher value of total tocopherols in all tested percentage of CS, especially the lowers ones. This higher amount in the second harvest can be due to a stress induced by calcium silicate over the longer period of time, provided that tocopherols are synthesized in response to stress situation as a product of the secondary or defensive metabolism of plants and mushrooms [30,31].

Table 3 shows the different individual fatty acids found in the two different strains, represented as relative percentages of total fatty acids. The table only shows the most abundant fatty acids, namely the ones with a relative percentage above 1%. Other fatty acids like C10:0 (decanoic acid), C11:0 (undecanoic acid), C12:0 (dodecanoic acid), C14:0 (tetradecanoic acid), C16:1 (hexadecanoic acid), C17:0 (heptadecanoic acid), C18:3n3 ((9Z,12Z,15Z)-octadeca-9,12,15-trienoic acid), C20:0 (eicosanoic acid), C20:1 (eicosenoic acid), C20:2 (Eicosadienoic acid), C20:5n3 ((5Z,8Z,11Z,14Z,17Z)-icosa-5,8,11,14,17-pentaenoic acid), C22:2 (docosanoic acid), C23:0 (tricosanoic acid), C24:0 (tetracosanoic acid) and C24:1 ((15Z)-tetracosenoic acid) were identified and quantified, but due to their very low amounts were not tabled and further discussed. Mushrooms have a very low amount of fat, and *Pleurotus ostreatus* var. florida is not an exception, the most abundant one is polyunsaturated, and thus the profile found in the studied mushrooms are consistent with the ones from literature, with C18:2n6c ((9Z,12Z)-octadeca-9,12-dienoic acid) being the most quantified, followed by C18:1n9c (octadec-9-enoic acid) and C16:0 (hexadecanoic acid) [27].

**Figure 2:**
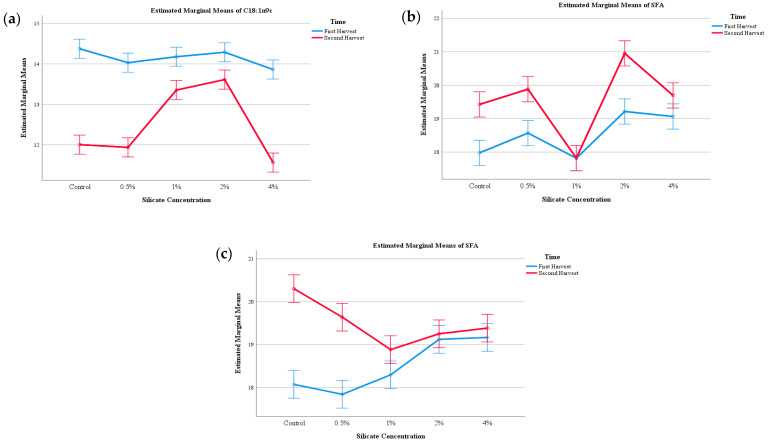
EMM plots of the Presidente Prudente mushrooms at different Si concentrations: (**a**) C18:1n9c (Octadec-9-enoic acid), (**b**) Saturated Fatty Acids (SFAs). EMM plots for Mogi-das-Cruzes: (**c**) SFAs.

As expected, the PUFAs (polyunsaturated fatty acids) accounted for over 66% of all fatty acids, which makes these mushrooms very healthy foods due to the beneficial effects of unsaturated fatty acids on human health. Saturated and monounsaturated fatty acids showed approximately 13 to 14 and 18 to 19%, respectively, which makes the saturated fat very low. Overall, the unsaturated fatty acids accounted for approximately 80% of the total. There was a significant interaction among both factors, HN and CS, and thus no individual classifications could be made, so, where possible some general conclusions were extracted from the EMM plots, shown in Figure 2, although the overall fatty acid profile of the mushrooms did not change much due to the supplementation of CS. Still, by analysing Figure 2, section (a) in the PP mushrooms, an interesting pattern can be found for oleic acid (common name, C18:1n9c), where despite variations in the second harvest, the amounts of this molecule was never as much as the ones found in the first harvest, indicating that the best mushrooms in terms of this unsaturated fatty acid are found in the first harvest, and this supplementation only affects the second harvest, although ever so slightly. Inversely, all saturated fatty acids are found in higher amounts during the second harvest, and thus, the first harvest shows lower amounts for all CS concentrations. Still, at 1% of silicate supplementation, there seems to be a very drastic reduction in SFAs (saturated fatty acids) (Figure 2b). Finally, regarding section (c) of Figure 2, pertaining to Mogi-das-Cruzes strain, a similar pattern is found, with higher amounts of SFAs in the second harvest. Unfortunately, no patterns could be found in the EMM for the unsaturated fatty acids.

Overall, it seems that the variations in fatty acids are more related to the passage of time than with supplementation of CS, with SFAs being higher in the second harvests and unsaturated ones in the first harvest. This pattern could be due to the freshness of the substrate and higher amounts of compounds the mushrooms can absorb, and thus have higher unsaturated fatty acids, whilst the second harvest, with a lower amount of available nutrients in the substrate, shows a higher amount of saturated fatty acids [32]. There seems to be very little effect of CS supplementation on the overall profile of fatty acids, with time and available nutrients being the main reason for the changes in the profile.

## 4. Conclusions

Very little is known in terms of the effects of CS on the chemical composition and biological efficiency of mushrooms. In this work, the impact of CS on the biological and chemical composition of oyster mushrooms was studied. CS supplementation did not lower production yields and seems to have very light positive effects in terms of the production of vitamin D_2_ and tocopherols. Interestingly, the harvest period, linked to the passage of time, seems to have a much deeper impact on the production yields (higher in the first harvest) and saturation of fatty acids.

## Figures and Tables

**Table 1:** Biological parameters of the two strains during the two harvest periods and different supplementation concentrations.

Presidente Prudente (PP)						
		Mushroom Weight (g)	Number of Mushrooms	Biological Efficiency (%)	Yield Ratio (%)	Productivity Rate (% per Day)
Harvest number (HN)	First flush	167 ± 52 *	50 ± 21 *	104 ± 32 *	8 ± 2 *	4 ± 1 *
	Second flush	110 ± 41	35 ± 16	69 ± 25	5 ± 2	2.0 ± 0.8
*p*-value (*n* = 10)	Student T test	<0.001	0.009	<0.001	<0.001	<0.001
Calcium silicate supplementation (CS)	Control	164 ± 65	40 ± 22	103 ± 41	8 ± 3	3 ± 2
	0.5%	147 ± 59	43 ± 16	92 ± 36	7 ± 3	3 ± 1
	1%	129 ± 45	47 ± 27	81 ± 28	6 ± 2	3 ± 1
	2%	124 ± 52	39 ± 21	77 ± 32	6 ± 2	3 ± 1
	4%	129 ± 48	43 ± 16	80 ± 30	6 ± 2	3 ± 1
*p*-value (*n* = 25)	Tukey’s HSD test	0.296	0.897	0.296	0.296	0.571
HN×CS (*n* = 50)	*p*-value	0.826	0.332	0.826	0.826	0.697
**Mogi-das-Cruzes (MC)**						
Harvest number (HN)	First flush	172 ± 27 *	46 ± 6 *	108 ± 13 *	9 ± 1 *	4 ± 1 *
	Second flush	135 ± 68	33 ± 4	84 ± 42	7 ± 2	2 ± 1
*p*-value (*n* = 10)	Student T test	0.044	0.031	0.044	0.044	<0.001
Calcium silicate supplementation (CS)	Control	174 ± 79	48 ± 30	108 ± 49	9 ± 4	4 ± 2
	0.5%	156 ± 43	35 ± 18	97 ± 27	8 ± 2	3 ± 1
	1%	159 ± 50	41 ± 12	100 ± 31	8 ± 2	3 ± 1
	2%	156 ± 44	43 ± 26	97 ± 27	8 ± 2	3 ± 1
	4%	124 ± 94	30 ± 19	78 ± 59	6 ± 5	3 ± 2
*p*-value (*n* = 25)	Tukey’s HSD test	0.529	0.350	0.529	0.529	0.708
HN×CS (*n* = 50)	*p*-value	0.455	0.264	0.455	0.455	0.430

In each row, for the Harvest number (HN), the asterisk (*) means different statistical differences among the two harvests, with an overall significance value of 0.05. The presented standard deviations were calculated from results obtained under different operational conditions. Therefore, these values should not be regarded as a measure of precision, rather as the range of the recorded values.

**Table 2:** Different bioactive compounds and soluble sugars detected in the two mushroom strains along the two harvests and CS supplementation concentrations.

Presidente Prudente (PP)											
		Ergosterol (mg/100 g)	Vitamin D_2_ (µg/100 g)	Fructose (g/100 g)	Mannitol (g/100 g)	Trehalose (g/100 g)	Soluble Sugars (g/100 g)	*α*-Tocopherol (mg/100 g)	*β*-Tocopherol (mg/100 g)	*γ*-Tocopherol (mg/100 g)	Total Tocopherols (mg/100 g)
Harvest number (HN)	First flush	107 ± 6	743 ± 133	0.16 ± 0.03	2.2 ± 0.2	19 ± 2	22 ± 2	0.002 ± 0.001	0.26 ± 0.03	0.05 ± 0.06	0.31 ± 0.07
	Second flush	107 ± 8	593 ± 73	0.2 ± 0.1	2.2 ± 0.6	15 ± 4	17 ± 4	0.002 ± 0.001	0.3 ± 0.07	0.06 ± 0.04	0.36 ± 0.05
*p*-value (*n* = 10)	Student T test	0.288	0.031	0.007	0.811	<0.001	<0.001	0.580	<0.001	<0.001	<0.001
Calcium silicate supplementation (CS)	Control	101 ± 8	496 ± 27	0.20 ± 0.03	2.8 ± 0.5	15 ± 3	18 ± 3	0.002 ± 0.001	0.32 ± 0.03	0.02 ± 0.01	0.34 ± 0.04
	0.5%	109 ± 6	658 ± 71	0.13 ± 0.03	1.8 ± 0.4	18 ± 3	20 ± 3	0.002 ± 0.001	0.24 ± 0.04	0.009 ± 0.004	0.25 ± 0.04
	1%	110 ± 1	734 ± 144	0.19 ± 0.08	1.91 ± 0.06	16 ± 8	18 ± 8	0.002 ± 0.001	0.34 ± 0.08	0.02 ± 0.01	0.36 ± 0.09
	2%	113 ± 1	779 ± 90	0.16 ± 0.03	2.0 ± 0.2	19 ± 1	21 ± 2	0.001 ± 0.0001	0.26 ± 0.01	0.13 ± 0.01	0.39 ± 0.01
	4%	101 ± 4	671 ± 89	0.2 ± 0.1	2.3 ± 0.2	18 ± 2	21 ± 2	0.001 ± 0.0001	0.23 ± 0.03	0.108 ± 0.004	0.34 ± 0.03
*p*-value (*n* = 25)	Tukey’s HSD test	<0.001	<0.001	0.006	<0.001	<0.001	<0.001	<0.001	<0.001	<0.001	<0.001
HN×CS (*n* = 50)	*p*-value	<0.001	<0.001	<0.001	<0.001	<0.001	<0.001	<0.001	<0.001	<0.001	<0.001
**Mogi-das-Cruzes (MC)**											
Harvest number (HN)	First flush	111 ± 6	899 ± 167	0.14 ± 0.03	1.9 ± 0.2	19±2	21 ± 2	0.003±0.001 *	0.2 ± 0.1	0.05 ± 0.03	0.25 ± 0.09
	Second flush	116 ±10	829 ± 175	0.18 ± 0.08	1.8 ± 0.4	17 ± 4	19 ± 4	0.0018 ± 0.0001	0.28 ± 0.06	0.04 ± 0.04	0.32 ± 0.05
*p*-value (*n* = 10)	Student T test	<0.001	<0.001	<0.001	0.062	<0.001	<0.001	0.009	<0.001	<0.001	<0.001
Calcium silicate supplementation (CS)	Control	113 ± 4	555 ± 51	0.2 ± 0.1	2.3 ± 0.3	18 ± 2	20 ± 2	0.0025 ± 0.0009	0.261 ± 0.005	0.013 ± 0.003	0.28 ± 0.05
	0.5%	123 ± 1	951 ± 90	0.16 ± 0.03	1.8 ± 0.4	17 ± 5	19 ± 6	0.0022 ± 0.0003	0.32 ± 0.08	0.011 ± 0.003	0.33 ± 0.08
	1%	100 ± 3	880 ± 50	0.15 ± 0.01	1.6 ± 0.2	16 ± 3	18 ± 3	0.002 ± 0.001	0.1 ± 0.1	0.04 ± 0.03	0.2 ± 0.1
	2%	117 ± 7	1022 ± 31	0.10 ± 0.02	1.63 ± 0.05	18 ± 2	19 ± 2	0.003 ± 0.001	0.23 ± 0.02	0.08 ± 0.02	0.31 ± 0.01
	4%	114 ± 6	913 ± 30	0.19 ± 0.06	2.0 ± 0.2	20 ± 2	22 ± 2	0.0014 ± 0.0007	0.25 ± 0.03	0.087 ± 0.006	0.34 ± 0.03
*p*-value (*n* = 25)	Tukey’s HSD test	<0.001	<0.001	0.006	<0.001	<0.001	<0.001	0.130	<0.001	<0.001	<0.001
HN×CS (*n* = 50)	*p*-value	<0.001	<0.001	<0.001	<0.001	<0.001	<0.001	0.058	<0.001	<0.001	<0.001

In each row, for the Harvest number (HN), the asterisk (*) means different statistical differences among the two harvests, with an overall significance value of 0.05. The presented standard deviations were calculated from results obtained under different operational conditions. Therefore, these values should not be regarded as a measure of precision, rather as the range of the recorded values.

**Table 3:** Fatty acids detected through GC-FID from the two mushroom strains across the two harvest periods and CS supplementation, expressed in relative percentage of themselves. Only fatty acids with a relative above 1% are tabled, although others were identified and quantified.

Presidente Prudente (PP)									
		C15:0	C16:0	C18:0	C18:1n9C	C18:2n6c	SFAs	MUFAs	PUFAs
Harvest number (HN)	First flush	1.4 ± 0.1	11.7 ± 0.4	2.8 ± 0.2	14.1 ± 0.3	65.8 ± 0.7	18.5 ± 0.6	14.6 ± 0.3	66.8 ± 0.7
	Second flush	1.7 ± 0.2	11.9 ± 0.5	2.7 ± 0.2	12.5 ± 0.9	66 ± 1	19 ± 1	13.0 ± 0.9	67 ± 1
*p*-value (*n* = 10)	Student T test	<0.001	0.025	<0.001	<0.001	0.002	<0.001	<0.001	0.006
Calcium silicate supplementation (CS)	Control	1.7 ± 0.3	11.7 ± 0.2	2.79 ± 0.06	13 ± 1	66.6 ± 0.7	18.7 ± 0.8	14 ± 1	67.6 ± 0.6
	0.5%	1.5 ± 0.4	11.8 ± 0.2	3.0 ± 0.1	13 ± 1	66.4 ± 0.4	19.2 ± 0.7	13 ±1	67.3 ± 0.4
	1%	1.3 ± 0.07	11.5 ± 0.2	2.7 ± 0.2	13.8 ± 0.4	66.9 ± 0.3	17.8 ± 0.1	14.3 ± 0.4	67.9 ± 0.4
	2%	1.59 ± 0.07	12.1 ± 0.8	2.80 ± 0.07	13.9 ± 0.5	64.3 ± 0.8	20 ± 1	14.5 ± 0.4	65.4 ± 0.8
	4%	1.6 ± 0.2	12.0 ± 0.6	2.65 ± 0.09	13.9 ± 0.5	66 ± 1	19.4 ± 0.5	13±1	67±1
*p*-value (*n* = 25)	Tukey’s HSD test	<0.001	0.001	<0.001	<0.001	<0.001	<0.001	<0.001	<0.001
HN×CS (*n* = 50)	*p*-value	<0.001	<0.001	0.001	<0.001	0.002	0.001	<0.001	0.001
**Mogi-das-Cruzes (MC)**									
Harvest number (HN)	First flush	1.29 ± 0.06	11.6 ± 0.3	2.7 ± 0.1	13.6 ± 0.6	66.4 ± 0.6	18.5 ± 0.6	14.1 ± 0.6	67.4 ± 0.6
	Second flush	1.6 ± 0.1	12.4 ± 0.3	2.8 ± 0.2	13 ± 1	66 ± 1	19.4 ± 0.5	13 ± 1	67 ± 1
*p*-value (*n* = 10)	Student T test	<0.001	<0.001	0.543	<0.001	0.045	<0.001	<0.001	0.146
Calcium silicate supplementation (CS)	Control	1.5 ± 0.1	12.2 ± 0.4	2.7 ± 0.1	13.9 ± 0.2	65 ± 1	19 ± 1	14.3 ± 0.2	66 ± 1
	0.5%	1.4 ± 0.2	11.9 ± 0.6	2.7 ± 0.3	13 ± 1	66.8 ± 0.5	18 ± 1	13 ± 1	67.7 ± 0.5
	1%	1.5 ± 0.3	11.9 ± 0.2	0.287 ± 0.03	13 ± 1	67.2 ± 0.8	18.6 ± 0.3	13.1 ± 0.1	68.3 ± 0.9
	2%	1.39 ± 0.07	12.3 ± 0.4	2.7 ± 0.1	12.5 ± 0.1	66.6 ± 0.3	19.2 ± 0.2	13.1 ± 0.1	67.7 ± 0.3
	4%	1.4 ± 0.1	11.9 ± 0.8	2.8 ± 0.1	14.0 ± 0.3	65.2 ± 0.8	19.2 ± 0.4	14.6 ± 0.4	66.1 ± 0.8
*p*-value (*n* = 25)	Tukey’s HSD test	<0.001	0.001	<0.001	<0.001	<0.001	0.001	<0.001	<0.001
HN×CS (*n* = 50)	*p*-value	<0.001	<0.001	<0.001	<0.001	<0.001	<0.001	<0.001	<0.001

C15:0—Pentadecanoic acid, C16:0—Hexadecanoic acid, C18:0—Octadecanoic acid, C18:1n9C—Octadec-9-enoic acid, C18:2n6c-(9Z,12Z)-octadeca-9,12-dienoic acid (IUPAC nomenclature). SFAs—saturated fatty acids, MUFAs—monounsaturated fatty acids, PUFAs—polyunsaturated fatty acids. In each row, for the Harvest number (HN). The presented standard deviations were calculated from results obtained under different operational conditions. Therefore, these values should not be regarded as a measure of precision, rather as the range of the recorded values.
